# Exploring the Critical Environmental Optima and Biotechnological Prospects of Fungal Fruiting Bodies

**DOI:** 10.1111/1751-7915.70210

**Published:** 2025-08-14

**Authors:** Amechi S. Nwankwegu, Sinang Hongsanan, Uzoma P. Nwankwegu, Ning Xie

**Affiliations:** ^1^ Shenzhen Key Laboratory of Microbial Genetic Engineering, College of Life Sciences and Oceanography Shenzhen University Shenzhen China; ^2^ Organization of African Academic Doctors Nairobi Kenya; ^3^ Guangdong Provincial Key Laboratory for Plant Epigenetics, College of Life Sciences and Oceanography Shenzhen University Shenzhen China; ^4^ Department of Food Science and Technology Ebonyi State University Abakaliki Nigeria

**Keywords:** critical environmental optima, fruiting body biotechnology: epigenetic regulation, fungal fruiting body development, multi‐omics

## Abstract

Fruiting body development is a principal mechanism in fungal morphogenesis, which often involves complex interplays of hormonal regulation, gene expression, and metabolic immobilisation influenced by environmental interactions, ultimately leading to the differentiation of multicellular structures. In fungal communities, including ascomycetes and basidiomycetes, fruiting body development ensures protection and facilitates the dispersal of ascospores. Constrained by environmental factors that vary across morphogenetic stages, a thorough synthesis of the critical ecological optima, which primarily regulate the multi‐omics footprint encompassing diverse molecular perspectives characterising fruiting body formations, is key. It underscores that exceeding the critical environmental ranges triggers dynamic shifts that adversely impact the fruiting body's eco‐resilience; however, operating below these optima is safer, as most fruiting body physiological activities are generally able to maintain normal functioning and stability, making the present study relevant to decision‐makers for optimal fruiting body commercialisation. It elucidates the recent advances in fruiting body biotechnologies, traversing agricultural/food, optimised cultivation strategies, environmental and health, bioactive compounds extractions, genetic engineering, and synthetic biology, promoting scalable bioproduction. Nonetheless, it proposes further studies emphasising omics‐driven strain‐substrate improvements/genomic modifications, incorporating CRISPR advances, to boost precision cultivation and enhance robust strain design. The study offers promising insights into complementing existing knowledge on fungal fruiting bodies and addresses challenges related to environmental complexity and uncertainties, aiming to drive sustainable industrial biotechnology.

## Graphical abstract

Mechanisms and strategies for fungal fruiting body development (FBD) are reported. The critical optima for the relevant environmental cues regulating FBD were synthesised. Fruiting body biotechnological applications and recent advances in designed approaches are elucidated. Impacts of the critical environmental factors in regulating the multi‐omics footprint during FBD are divulged. Emerging CRISPR‐Cas9 technologies are strategically oriented to address the existing CRISPR limitations.

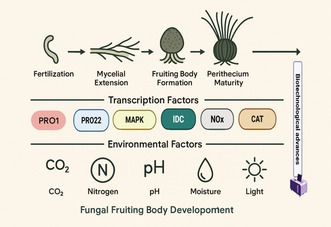

## 1 Introduction

Most fungal taxa form characteristically protruding, multicellular, and thread‐like structures called hyphae, which develop into complex, interconnected networks of high cell density known as mycelia (Meletiadis et al. 2001; Meyer et al. 2021), representing the essential bodies of the fungi. As a key integrant of the soil microbiome, they decompose organic matter as saprotrophs. However, they are ubiquitous and sometimes occur as laboratory contaminants in culture plates. Some common fungal communities include Mucor, *Rhizopus* spp., *Podospora* spp., *Penicillium* spp., *Neurospora* spp., *Aspergillus* spp., *Fusarium* spp., *Scedosporium* spp., as well as *Trichoderma* spp., *Acremonium* spp., *Scopulariopsis* spp., *Purpureocillium* spp., and *Paecilomyces* spp. Fungi show immense economic importance and are known for their nutritional, industrial, and medicinal significance (Ferreira et al. 2020; Żukiewicz‐Sobczak et al. 2015). Fungal commercial cultivation or survival in the natural environment often requires selective responses to critical environmental parameters, which are usually controlled by specialised cellular regulatory networks to initiate sexual reproductive development, known as the fruiting body. Controlled environment cultivation (CEC) and suitable substrate management are crucial for establishing optimum conditions for enhanced fruiting body development (Lin et al. 2022; Pavlík et al. 2020).

**FIGURE 1:**
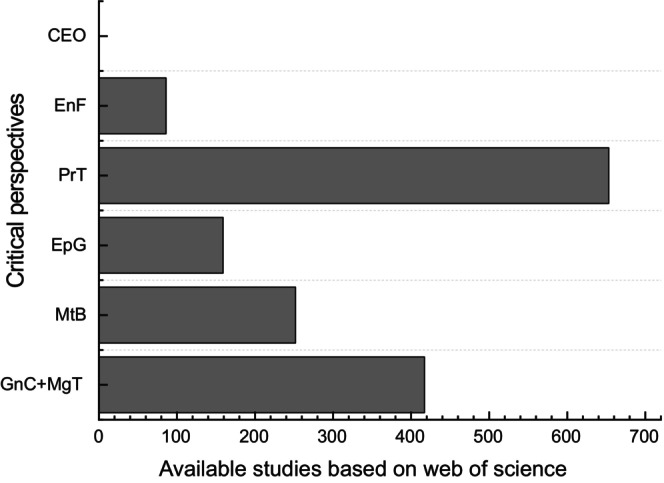
Proportion of the available literature based on Web of Science core collections on fungal fruiting body development, which involved the potential omics footprint and associated environmental cues. GnC + MgT (417) = Genomics + Metagenomics, MtB (252) = metabolomics, EpG (159) = Epigenetics, PrT (653) = Proteomics, EnF (86) = Environmental factors, and CEO (0) = Critical environmental optima. Studies that emphasised critical environmental optima have not been documented, necessitating the present study.

Fungal fruiting body development has gained considerable attention in recent years; this is especially true for basidiomycetes (Lyu et al. 2021; Nagy et al. 2011; Nowrousian 2018; Pent et al. 2020; Sakamoto 2018; Song et al. 2018) and several ascomycetes (Cai et al. 2021; Hao et al. 2019; Kikuma et al. 2006; Nowrousian 2018; Wang, Wang, et al. 2019; Wang, Xiao, et al. 2019), which are multicellular. Although ascomycetes and basidiomycetes mostly produce fruiting bodies during various sexual developmental stages, they show considerable differences in their evolutionary adaptations and ecological niches (Wen et al. 2023). Specifically, ascomycetes produce spores inside asci, while basidiomycetes produce spores externally on basidia. Again, the ascomycetes exhibit diverse fruiting bodies, such as apothecia and perithecia, while basidiomycetes usually form mushrooms and brackets (Sakamoto 2018). Furthermore, ascomycetes are diverse in niches and functions, including symbionts and plant pathogens, while basidiomycetes are often associated with parasitism, mycorrhizal associations, and wood decay. The fruiting body development is a complex process encompassing genetic, physiological, and chemical signalling molecules to mediate environmental stress interactions (Wen et al. 2023). The optimal fruiting body development is often dependent on several critical environmental signals. Research evaluating the impacts of environmental factors on fruiting body development is a dynamic field, intensified by rapid technological advancement and a growing appreciation of fungal biology. Presently, a plethora of omics studies on fruiting body development have been documented, including molecular mechanisms and genetic regulations, reflecting genomic and transcriptomic protocols (Chen et al. 2024; Hao et al. 2019; Kikuma et al. 2006; Lyu et al. 2021; Meng et al. 2021; Nagy et al. 2011; Nowrousian 2018; Pent et al. 2020; Shen et al. 2024; Song et al. 2018; Wang, Xiao, et al. 2019; Zhang et al. 2018), proteomic and metabolomic apparatuses elucidating crucial proteins and secondary metabolites from fruiting bodies (Chen et al. 2024; Domingo et al. 2023; Li et al. 2023; Verma et al. 2024; Xie et al. 2024; Zhao et al. 2024), epigenetic and developmental plasticity to track post‐transcriptional/translational modifications (Bertossa et al. 2004; Grube and Wedin 2017; Ribeiro et al. 2024; Schindler and Nowrousian 2014; Vonk and Ohm 2018), microbiome interactions, particularly ectomycorrhiza and endosymbionts (Berrios et al. 2023; Blackwell and Vega 2018; Grube and Wedin 2017; Imamura and Yumoto 2008; Pham et al. 2012; Ugawa et al. 2009; Verma et al. 2024), eco‐physiological and biotic interactions (Boddy et al. 2014; Tsujino et al. 2009), computational modelling and system biology for predictions of fruiting body interactions (Bailly 2023; Jo et al. 2023; Kijpornyongpan et al. 2022; Meškauskas et al. 2004; Moore and Stočkus 1998; Ogawa and Yashima 2023; Wang et al. 2018; Wu et al. 2022), and the applications of biotechnology for uncovering eco‐friendly, cost‐effective, and sustainable cultivation practises, as well as alternative substrates assessment for commercial bioproduct scale‐ups (AbuQamar et al. 2023; Afifa et al. 2022; Elnahas et al. 2024; Jo et al. 2023; Lazur et al. 2024; Pepe et al. 2023; Xu et al. 2023; Zhao et al. 2024). Despite all these research breakthroughs, the critical optimum for the essential environmental factors that influence fruiting body development remains largely unclear. The critical environmental optima define the boundary beyond which a slight drift in essential environmental factors can significantly impact fruiting body formation, warranting a dramatic shift. Understanding the critical environmental ranges is also crucial for making informed decisions and predicting potential risk levels associated with specific environmental signals that drive fruiting body development. These studies, which divulged omics perspectives, are undoubtedly robust and crucial; however, considering the huge variability in fungal diversity and their heterogeneity in recognising environmental stimuli, reports on the critical environmental optima influencing these molecular cues are important and require adequate exploration. Indeed, a few available studies on the potential implications of environmental factors on fruiting body development, although they recognised the variabilities in responses to environmental factors, did not capture their critical optima. Specifically, Sakamoto (2018) ascertained the influence of environmental factors on fruiting body induction, development, and maturation in basidiomycetes, and qualitatively emphasised the effects of temperature, nutrients, and light. Again, Wen et al. (2023) evaluated the potential effects of heat stress and excessive maturity of the fruiting body on a health‐promoting bioactive metabolite known as Gamma‐aminobutyric acid (GABA) in the oyster mushroom (*Pleurotus ostreatus*) and reported suppressions of GABA accumulation and downregulation of GABA genes by increases in heat stress and excessive maturity. Hence, the present study aims to synthesise these critical environmental optima that potentially drive optimum fungal fruiting body development. In addition, it explores the recent advances in fruiting body biotechnology to boost fruiting body growth and yields. To the best of our knowledge and strongly supported by thorough literature searches, as shown in Figure 1, the present study is the first to report the critical environmental optima driving fruiting body development in ascomycetes and basidiomycetes. The study is promising, as filling this knowledge gap would substantially complement existing literature on fruiting body development, optimise fungal biotechnological prospects, and largely circumvent environmental heterogeneity and sustainability challenges.

## 2 Environmental Cues and Their Critical Optima

The critical environmental optima refer to the specific conditions in ecological stressors that could trigger significant changes in the functioning of fruiting bodies once exceeded (Karavani et al. 2018). Most times, being below the critical optima could potentially trigger resource scarcity, such as water or nutrient limitations, and adversely impact ecosystem services and fungal health (Karavani et al. 2018). However, rather than exceeding the critical optima, operating below it is safer for the fruiting body performance, considering that they can generally maintain relative functioning and stability. Nevertheless, it is pertinent to consider specific circumstances and species, as different fruiting bodies exhibit considerable variabilities in their eco‐tolerance levels (Chadwick and Lin 2024). A thorough understanding of these critical environmental optima is thus crucial for large‐scale fruiting body cultivations. It determines the vitality, viability, functioning, and health of fruiting bodies. It offers evidence‐based support to researchers and policymakers to make informed decisions towards promoting sustainable fruiting body production for various applications. The critical environmental optima for enhanced fruiting body development are thus enumerated, as shown in Table 1.

**TABLE 1:** Critical environmental optima for optimal fungal fruiting body development.

Environmental factor	Optimum range	Critical optima
Aeration		
CO_2_ ^a^	10,000–20,000 ppm	15,000 ppm (spawning)
		1000 (early stage)
Dissolved O_2_ ^a^	2.0–2.8 mg/L	2.8 mg/L (aerobic condition)
	1.0–1.2 mg/L	1.2 mg/L (hypoxic condition)
Humidity	80%–95%	95%
Nutrient		
Nitrate	10–45 mg/L	45 mg/L
Phosphorus	0.2%–0.5%	0.5% (FFB dry weight)
Potassium	10–30 mg/g	30 mg/g
Temperature	15°C–27°C	25°C
Light	10–2000 lux (1–200 μmol/m^2^/s)	2000 lux (200 μmol/m^2^/s)
Wavelength		
Blue light	400–500 nm	500 nm
Red light	600–700 nm	700 nm
pH	4.5–7.5	7.5

Abbreviations: CO_2_, carbon (IV) oxide; FFB, fungal fruiting body; O_2_, oxygen.

^a^ Refers to indices that show variable effects under certain conditions.

### 2.1 Aeration

Appropriate air exchange is crucial to prevent harmful gas accumulations and promote the diffusion of metabolic gases, including carbon (IV) oxide (CO_2_) and oxygen (O_2_). Elevated CO_2_ levels can trigger fruiting body development in most fungi, while further developmental stages require lower levels. The CO_2_ concentrations between 10,000–20,000 ppm during the spawning and not ≥ 1000 ppm, preferably between 500 and 800 ppm during the fruiting body stage, are usually recommended. Specifically, Sakamoto (2018) reported that high CO_2_ concentrations can pre‐empt fruiting body formation by suppressing the effective pileus differentiation in basidiomycetes. Proteins associated with hydrolase activity, such as amidohydrolases and those involved in cell wall synthesis, have been highly expressed under high CO_2_ concentrations; however, kinases and elongation factors were significantly downregulated under the same condition, indicating that high CO_2_ potentially inhibits metabolic regulation (Lin et al. 2022). The CO_2_ directly interacts with regulator proteins, e.g., Ptc2, initiating phase separation and inducing signal transduction by directing protein localisation and post‐translational modifications (Chadwick and Lin 2024). In mushrooms, CO_2_ concentration is highly relevant during primordia formation, when the mycelium begins to differentiate to form characteristically tiny initial structures generally referred to as pins that eventually mature into typical fruiting bodies; hence, this process is called pinning (Sakamoto 2018). The sensitivity and/or tolerance to CO_2_ is intensified during the early stage of fruiting body development (Sakamoto 2018). Similarly, a previous study evaluated the CO_2_ tolerance in the mutant strain HY68 of *Hypsizygus marmoreus*, an edible mushroom native to East Asia, which can maintain a normal fruiting body under high CO_2_ levels and reported superior CO_2_ tolerance in the HY68 mutant (Liu et al. 2023). Furthermore, Pavlík et al. (2020) estimated mycelial respiration of two basidiomycetes (*Pleurotus ostreatus* and *Ganoderma lucidum*) and reported metabolically demanding and higher CO_2_ flux during the early mycelial growth stage. As for O_2_ concentration, most fungi are obligate aerobes, showing optimal fruiting body development at an O_2_ concentration of < 1%. The dissolved oxygen (DO) availability during fruiting body development varies strongly depending on the species type and dominant environmental conditions; nonetheless, critical optima usually range from 2 to 8 mg/L under aerobic and 1–2 mg/L under hypoxic conditions for facultative aerobes. An absence of O_2_ potentially halts primordia development into a fruiting body (Zain Ul Arifeen et al. 2021). In other words, any environmental disturbance that causes a drop in O_2_ levels (DO ≤ 0) could threaten fruiting body development and shift morphogenesis to anaerobic metabolic pathways. Similarly, Zain Ul Arifeen et al. (2021) studied the effect of O_2_ on fruiting body development in the sub‐seafloor fungus *Schizophyllum commune* 20R‐7‐F01 and reported a minimum oxygen concentration requirement of 0.5% pO_2_ to spawn primordia and 1% pO_2_ to transform primordia into a mature fruiting body. A previous study also ascertained the oxygen permeability of intact fruiting body pipelines/skins among five species of basidiomycetes, including *Amanita*, *Russula*, *Stropharia*, *Tapinella*, and *Tricholomopsis*, and reported lower O_2_ permeability, which ranged from 0.8 to 6.0 × 10^−6^ ms^−1^. Based on the study, water and oxygen permeability depend on ecosystem humidity, indicating that pipeline permeability increases with higher humidity in the air (Lendzian and Beck 2021). However, an inhibitory effect has been reported on the fruiting body following the dissolved O_2_ reduction below 0.8 volumes of oxygen at standard temperature and pressure (Miller and Golding 1949).

### 2.2 Humidity

This technically refers to the amount of water vapour in the air and is a crucial physical/environmental parameter for most biological processes. High humidity levels are necessary for fruiting body development, and the relative humidity levels for fruiting body development range between 80% and 95% for most ascomycetes and basidiomycetes. Fungal fruiting body development is promoted by humid conditions (Cikarge and Arifin 2018), indicating that yield primarily depends on climate change indices, including weather and soil moisture conditions (Karavani et al. 2018). The substrate composition and moisture content are critical, and the optimum substrate requirements for effective fruiting body development are nutrient richness and adequate hydration. The woods and soil are suitable substrates; however, several modified materials with high lignocellulosic contents, including rice straws/husks, wheat bran, wood chips, sawdust, sugarcane bagasse, and palm oil wastes (shaft and bunch), are frequently utilised as suitable substrates for fruiting bodies (Elkanah et al. 2022; Hoa et al. 2015), promoting cost‐effective and sustainable industrial bioproduction.

### 2.3 Nutrients

The availability of essential nutrients in the substrate, including nitrogen, phosphorus, potassium, and high‐energy carbon sources like glucose, is one of the crucial fruiting body triggers. The nitrate concentration of 10 mg/L is sufficient to drive optimum fruiting body development, with a critical optimum of 45 mg/L. Incorporation of nitrate into the nutrient regimen helps stimulate growth, and the arbuscular mycorrhizal (AM) fungi play a crucial role in maintaining ecosystem stability and functioning (Wu, Uchida, et al. 2023; Wu, Yang, et al. 2023; Xu et al. 2022). However, excessive N enrichment can adversely impact fruiting body formation (Sakamoto 2018; Xu et al. 2022). Phosphorus (P) and potassium (K) are also crucial for disease resistance, fruiting body hardiness, cellular homeostasis, and osmoregulation (Kamal et al. 2012). The critical concentrations P (0.2%–0.5% of the fruiting body dry weight) and K (10–30 mg/g dry weight) are ideal for fruiting body development. The soil P availability is generally low (Etesami et al. 2021) with the normal soil P and K in fungal‐soil ecosystems ranging between 11.0–22 and 10–280 kg/ha, respectively, indicating why fungal biomass of soil saprotrophs usually significantly shows high P concentration relative to the ectomycorrhizal and wood saprotrophic fungi (Pánek et al. 2024). The phosphorus uptake mechanism by the fruiting body is usually by the secretions of hydrolytic enzymes (Meeds et al. 2021). Kamal et al. (2012) evaluated the effect of P enrichment based on the mycelial growth rate, substrate utilisation, and production of lignocellulosic extracellular enzymes on four different fungal species/strains, including *Agaricus bisporus* (S‐11), *A. bisporus* (U‐3), *Pleurotus florida* (P‐1), and *Volvariella volvacea* (OE‐12). They reported increased mycelial growth rates (doubled), protein production, and 4–5‐fold increases in extracellular enzymes. Furthermore, the optimum concentrations of P for mycelial growth were established at 0.1%, 0.05%, and 0.025% fo*r V. volvacea, A. bisporus*, and *P. florida* , respectively. Physiological responses significantly increased upon K‐ and P‐amendments (Rosenstock et al. 2016). Moreover, other elements, including sulphur (S), magnesium (Mg), calcium (Ca), molybdenum (Mo), and gallium (Ga), as well as some trace elements like iron (Fe), zinc (Zn), manganese (Mn), and copper (Cu), are also needed to offer stimulatory effects on fruiting body development; however, their specific impacts on fruiting body development require further studies. Regarding glucose uptake as a major carbon (C) source, fruiting body development decreases under higher glucose gradients. Sakamoto (2018) posited that low C can trigger fruiting body formation, while media containing elevated C can suppress fruiting body development. The putative starvation‐induced pathways, including G‐protein‐coupled receptors (GPCRs), Ras, cAMP‐dependent pathways, MAPK, nitrogen, and carbon sensing pathways, are crucial for fruiting body adaptations to oligotrophic ecosystems. The cAMP‐dependent pathway activates nutrient sensing and chemotactic cell aggregation in most fruiting bodies. Glucose supply boosts cAMP gradient under starved cell conditions by activating adenylate cyclase to cause binding between cAMP and cAMP‐dependent protein kinases (PKAs), thus initiating mating and sporulation. However, reduced cAMP and PKA activity induce sexual development and produce fruiting bodies as a direct response to starvation stress (Virágh et al. 2022). Again, nitrogen starvation was reported to induce the differentiation of protoperithecium into perithecium in *Neurospora* spp. (Colot et al. 2006). Caloric restriction or nutrient limitation promotes the differentiation of aerial hyphae into mature fruiting bodies (Colot et al. 2006). Typically, *Podospora anserina* demonstrates optimum growth on a medium not too rich in digestible nitrogen and carbon sources (e.g., ammonium and glucose) as it produces large amounts of mycelia, especially aerial hyphae that remain sterile or could reproduce but are delayed with abnormal perithecia and a few asci under rich‐medium incubation (Silar 2005). In other words, a high glucose concentration can impose a metabolic burden that hinders overall morphogenetic activities, initiates osmotic stress, and disrupts cellular homeostasis, ultimately impacting fruiting body viability. Again, elevated glucose concentration could lead to cell toxicity and downregulation/inhibition of pathways associated with alternative carbon utilisation through catabolite repression of carbon. In nutrient‐replete ecosystems, organisms employ the mechanism of catabolite repression to preferentially utilise alternative nutrient sources with characteristically lower energetic burdens, thereby circumventing the metabolic cost. A typical example is the frequently reported preference for ammonium (NH_4_^+^‐N) over nitrate NO_3_^−^‐N metabolisms (Li et al. 2020; Nwankwegu et al. 2020; Tudzynski 2014) since NH_4_^+^‐N, a lower oxidation state of N‐form, is easily incorporated into amino acids unlike NO_3_^−^‐N, which has to be first broken down to NH_4_^+^‐N prior to assimilation.

### 2.4 Temperature

Temperature is an important environmental factor influencing fruiting body development, from initiation to perithecia maturation and spore dispersal (Sakamoto 2018). Knowledge of the specific temperature requirements and tolerance levels of fruiting bodies is essential. Although different fruiting bodies have specific temperature requirements for effective sexual development, critical temperatures between 15°C–27°C are generally optimal for fruiting body formation. Similarly, Silar (2020) estimated growth dynamics under different temperature conditions and posited that perithecium maturity occurs within 3–4 weeks on M2‐mycelium, approximately 9–10 days at 23°C, and 7 days at 27°C. At temperatures above 29°C–30°C, no fruiting body was observed. Although mycelial growth can proceed up to 37°C, a potential heat shock at 37°C has been reported in the coprophilous ascomycete *P. anserina* (Silar 2020). Relatively robust fruiting body structures are associated with temperature downshift, while a higher temperature regime can result in malformed fruiting body structures (Sakamoto 2018). Nonetheless, being too high or cold would adversely affect fruiting body development, altering osmoregulatory, metabolic, and protein pathways (Silar 2020). The warmer temperature is crucial for spore dispersal, as often seen in the force of discharge in ballistospores‐producing fruiting bodies (Liu, Chang, et al. 2017; Liu, Chavez, et al. 2017; Money 2023; Pringle et al. 2005). The ascomycetes and basidiomycetes adapted to cold ecosystems can potentially form fruiting bodies in high‐altitude or high‐latitude areas. In contrast, those adapted to warm conditions are typically found in tropical or subtropical regions. Again, in temperate zones, fruiting bodies usually form in the spring and fall when temperatures are relatively mild, whereas in tropical areas, fruiting body formation may be continuous and occur year‐round or during cooler periods (Krah et al. 2023). Although some fruiting bodies have developed mechanisms to cope with fluctuations in temperature, including the production of antifreeze proteins (AFPs) or heat‐shock proteins (HSPs), allowing them to thrive and maximise fruiting body formation under less‐than‐ideal conditions (Bakar et al. 2020; Tiwari et al. 2015). In certain situations, the indirect effect of temperature may depend on its interactions with other environmental factors, such as nutrient availability, humidity, and light. For example, a specific temperature may be optimal for inducing fruiting body formation only when sufficient nutrients, high humidity, or adequate light are available.

### 2.5 Light and Gravity

Light, measured in intensity (lux or μmol/m^2^/s) rather than concentration, can positively impact fruiting body development by influencing the initiation and direction of growth; however, some species may require darkness or specific light conditions to thrive. Depending on the fruiting body species, the optimum light intensity for the fruiting body ranges from near darkness (0 lx) to moderately high light levels (10–2000 lx or 1–200 μmol/m^2^/s). Despite the variabilities, the optimum intensity remains 2000 lx (200 μmol/m^2^/s). Aside from intensity, the duration and wavelength of light exposure are also crucial, as they act in tandem to impact the fruiting body formation. For example, (1) blue light with a 400–500 nm wavelength is particularly effective in fruiting body induction and regulates most of the fruiting body developmental processes. (2) Red light, with a wavelength of 600–700 nm, is generally less effective when acting alone, although it usually interacts with blue light to influence fruiting body development. Light enhances pileus differentiation, facilitating effective spore diffusion, but it also affects the thickness and length of the stipes. Stipe elongation occurs in the dark, while it thickens in the light (Sakamoto 2018). Fruiting bodies possess light‐regulatory networks (Cerón‐Bustamante et al. 2023; Schumacher 2017; Yu et al. 2023), which can sense light through flavin‐based proteins, such as rhodopsin (Cerón‐Bustamante et al. 2023). On the other hand, gravity influences the direction and orientation of fruiting bodies. The ability of fungi to respond effectively to gravity through coordinated mechanisms is known as gravitropism. The clinostat and space‐flown experiments previously demonstrated that the basic form of the mushroom, including the overall tissue organisations of stem, gills, cap, veil, and hymenium, is established independently of the gravity vector; however, maturation, and particularly processes leading to the meiosis–sporulation pathway, require the conventional gravity vector (Moore 1996). Over the years, dramatic changes have occurred as gravity can ensure fruiting body development along environmentally safe and strategic directions for spore dispersal. Most basidiomycetes show obligate phototropism and negative gravitropism for effective spore dispersal (Sakamoto 2018). Similarly, Moore (1996) reported that fruiting body primordia are usually positively phototropic and negative gravitropic. Generally, switches between tropisms are linked to meiosis, and the mycelia, which grow downwards into the underlying substrate, usually show positive gravitropism.

As for pH, the optimal pH condition for fruiting body formation often varies considerably depending on the species; nonetheless, most fungal fruit bodies potentially exhibit optimal activity in a slightly acidic to neutral pH range (Shim et al. 2005). Depending on the species, the pH of the fruiting body can range from 4.5 to 7.5. The pH conditions, including *Agaricus bisporus* (pH = 6.0–7.5), *Pleurotus ostreatus* (pH = 5.0–7.5), and *Lentinula edodes* (pH = 4.5–6.5), are optimal for fruiting body development in the basidiomycetes. Meanwhile, the optimal pH for most ascomycetes ranges from 5.5 to 7.0. Similarly, optimal mycelial growth of *Phellinus* spp. at pH 6–7 has been previously reported (Hur et al. 1971). Despite the fruiting body species, the critical pH optimum for fruiting body development does not exceed 7.5 except for the ascomycetes, *Tuber* spp. (e.g., Tuber affine, *Tuber aestivum, Tuber borchii*, etc.) that thrive in the pH range of 7.5–8.5, considering their affinity to slightly alkaline conditions. The pH is a critical factor that impacts the fruiting body processes, including nutrient availability, reproductive efficiency, substrate decomposition, enzyme activity, and structural integrity. The pH range of 5.0–6.5 can favour a wide range of fungal cell factories, particularly biotechnologically relevant species like *Penicillium* spp., *Aspergillus* spp., *Trichoderma* spp., *Rhizopus* spp., *Fusarium* spp., *Yarrowia* spp., *Mucor* spp., and *Ganoderma* spp. The diverse biotechnological applications of fungal fruiting bodies in pharmaceuticals, nutraceuticals, biofuels, food production, and environmental sustainability are outlined in Table 2.

**TABLE 2:** Biotechnologically relevant fungal fruiting body species, products, and potential applications.

Species^a^	Biotechnological application	Product	Use	References
*Ganoderma lucidum*	Nutraceuticals/Medicinals	Triterpenoids and polysaccharides	Traditionally used for immune‐boosting, anti‐ ageing, and anticancer	Azi et al. (2024)
*Pestalotiopsis microspore*	Bioremediation	Plastics waste management	Polyurethane degradations	Hawkins et al. (2023)
*Cordyceps militaris*	Medicine	Cordycepin and polysaccharides	Bioactive supplements	Shweta et al. (2023)
*Mucor indicus*	Organic solvent/Polymer productions	Ethanol/Chitosan	bioethanol production, biocontrol agriculture, and water treatments	Zhou et al. (2020)
*Yarrowia lipolytica*	Bioplastic and lipid productions	Polyhydroxyalkanoates (PHAs) citric acid, and lipids	Green energy production (lipids conversion to biodiesel and bioplastics) which are biodegradable substitutes to petroleum‐derived plastics.	Shweta et al. (2023)
*Rhizopus oryzae*	Organic acid and fermentation production	Fumaric and lactic acids	Pharmaceuticals and nutraceuticals (as food additives)	Zhou et al. (2020)
*Fusarium venenatum*	Protein production	Mycoprotein	Meat substitutes referred to as Quorn	Ghorai et al. (2009)
*Trichoderma reesei*	Industrial enzyme production	Cellulases and hemicellulases	Cellulases used for industrial conversion of cellulose to glucose in biofuel production, animal feed, textile, and paper	Islam et al. (2023)
*Aspergillus oryzae*	Fermentation and enzyme production	Soy sauce, proteases, and amylases	Used as food production enzymes and as fermented soybeans (e.g., sauce and miso)	Ghorai et al. (2009)
*Saccharomyces cerevisiae*	Fermentation and bioethanol production	Ethanol	As bakers' yeasts for baking, brewing and bioethanol production, ethanol and carbon dioxide from sugar fermentation.	Amara and El‐Baky (2023)
*Aspergillus niger*	Enzymes production	Phytase, citric acid, glucoamylase and pectinase	Food and beverage industries for citric acid production animal feed enhancement, preservatives, flavour enhancer, starch processing enzymes, and fruit juice clarification	Ghorai et al. (2009)
*Penicillium chrysogenum*	Antibiotic production	Penicillin	Narrow‐spectrum antibiotic for treatment against bacterial infections	Rousta et al. (2024)

^a^ Fungal fruitbodies perform crucial roles in industrial and environmental biotechnology, considering their ability to produce various antibiotics, bioactive materials, and enzymes.

## 3 Mechanisms and Stages of Fruiting Body Development

**FIGURE 2:**
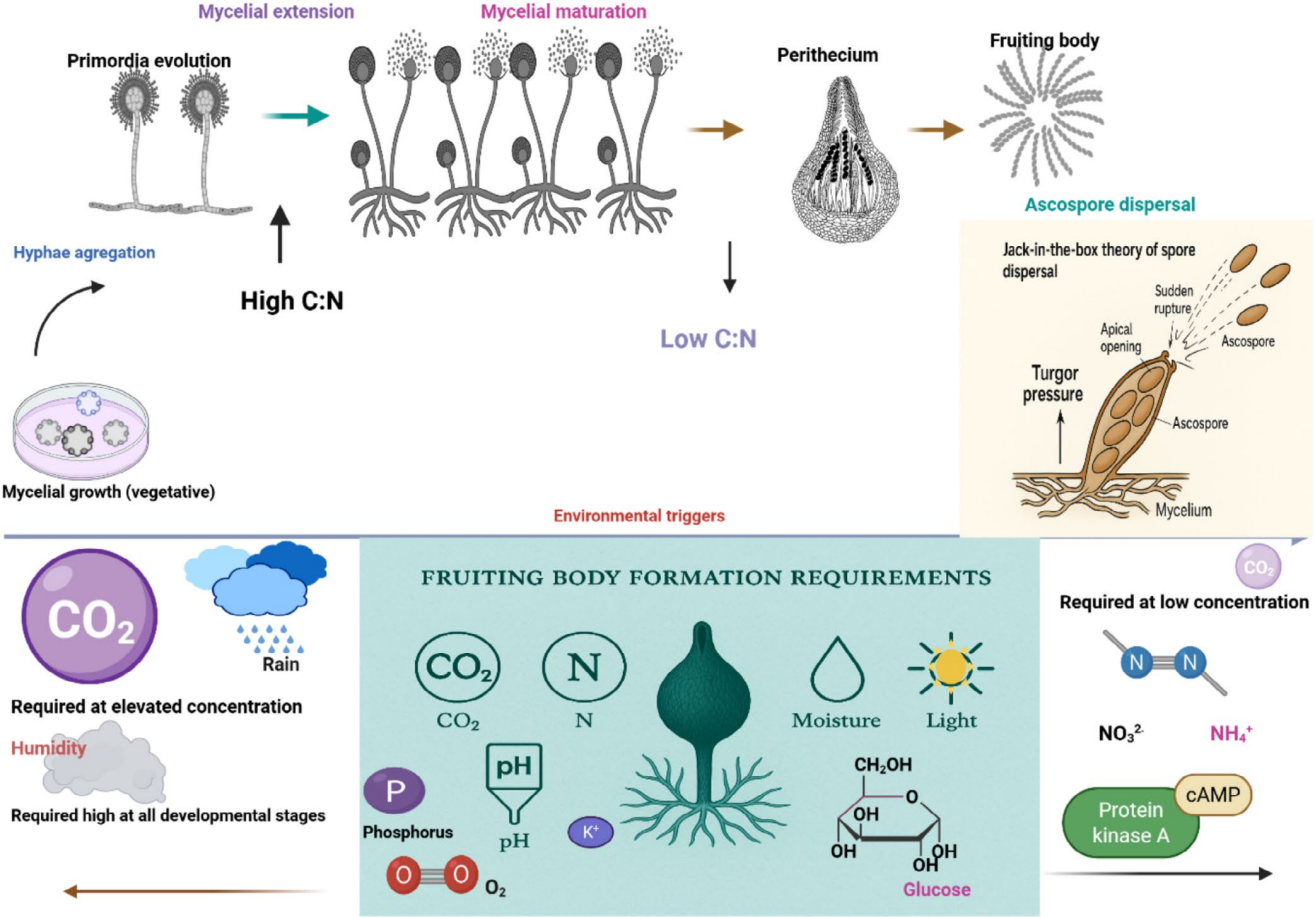
Fungal fruiting body development processes from the hyphae aggregation to perithecium maturity and their crucial stage‐dependent triggers/environmental requirements.

Fruiting body development generally involves complex mechanisms controlled by multiple interactions, including environmental signals, physiological, biochemical, and genetic regulation, as well as cellular differentiation (Sánchez‐García et al. 2020). The fruiting body mechanisms extensively provide insights into the processes that often involve transitioning from vegetative growth to sexual reproductive development, allowing fungi to produce spores for dispersal upon maturity. Despite the remarkable advancements in studies involving mechanisms of fruiting body formation, numerous aspects still require exploration, creating potential research avenues for further investigation. Fruiting body development usually consists of five key stages (Figure 2) in tandem with environmental triggers (adjustments in nutrients, temperature, light, humidity, etc.), genetic regulations (master regulatory genes and signal transduction pathways), as well as fruiting body diversity (Sánchez‐García et al. 2020). The stages centrally include.

### 3.1 Hyphal Aggregation

**FIGURE 3:**
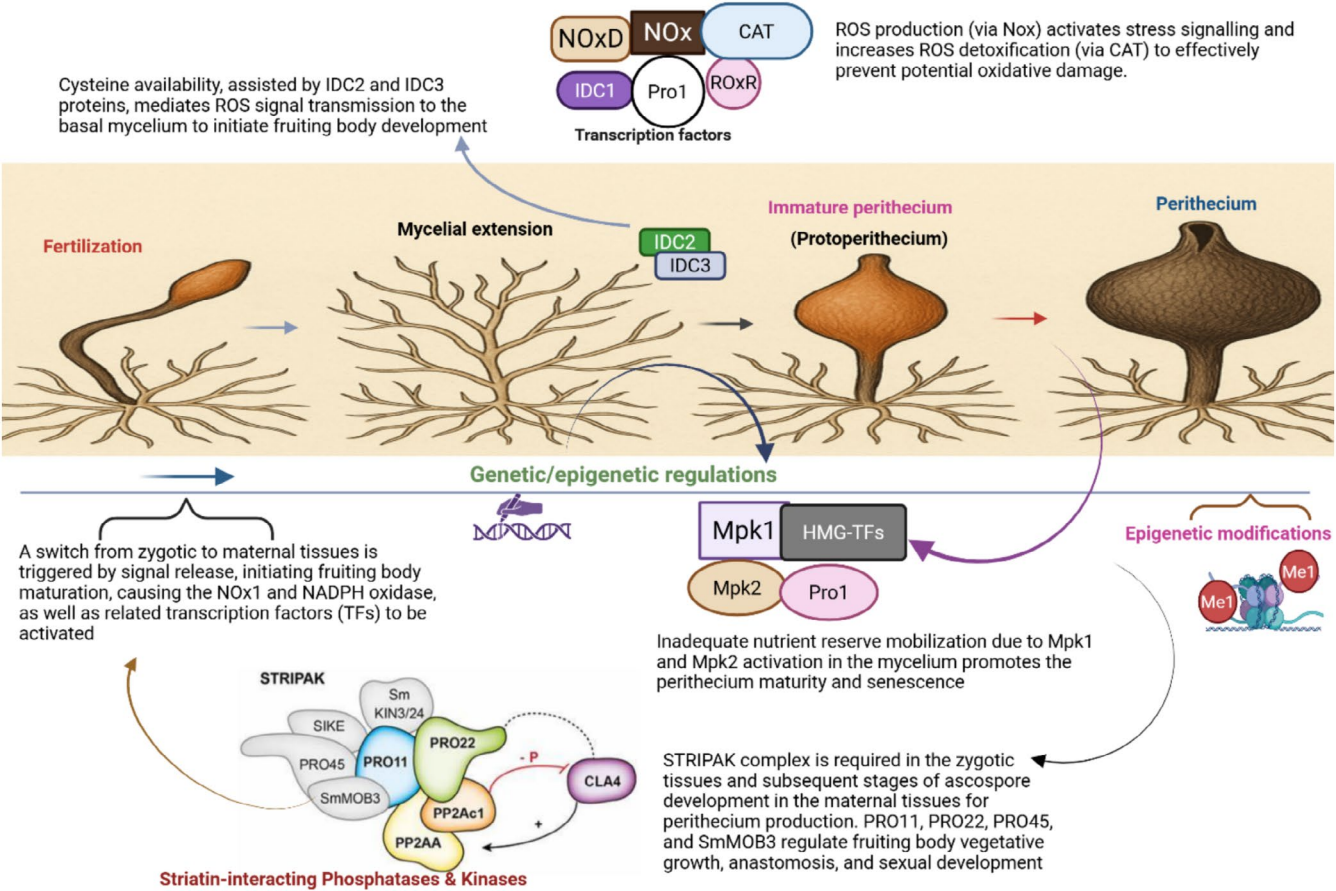
Perithecium development processes and the functional transcription factors (TFs) regulating fruiting body development. The TFs are involved in neck differentiation, and their targeted knockouts usually promote spore dispersal. IDC1 and Pro1 play crucial roles in the maternal tissues to ensure proper development. The striatin‐interacting phosphatases and kinases (STRIPAK) complex regulates signal transduction and fruiting body development. It potentially interacts with the mitogen‐activated protein kinase (MAPK) Mgv1, a crucial cell wall integrity pathway to regulate the phosphorylation dynamics and MgV1 nuclear accumulation, thereby controlling the fungal fruiting body stress responses.

This is the first stage and often involves transitioning from vegetative growth to sexual reproductive growth induced by physiological changes. A set of cell surface proteins, including hydrophobins, offers crucial functions in the aerial growth of hyphae. Specifically, studies have reported that hydrophobin‐SC3 in *Schizophyllum commune* possesses 16–22 O‐linked mannose residues and potentially binds to the N‐terminal peptide chain in the cell wall of monokaryotic and dikaryotic hyphae (de Vocht et al. 1998; Virágh et al. 2022). Hyphal aggregation is one of the fruiting body response mechanisms to environmental cues, usually characterised by anastomosis between adjacent hyphae (Virágh et al. 2022). In *P. anserina*, for example, > 80% ascospore germination is induced when ascospores are predisposed to some anthropogenic stressors, including exposure to the chemical environment like phenolic compounds (10^−3^–10^−4^ M), H_2_O_2_ at 3% for 20 min, pancreatin activity for 1–2 h at 37°C, and physical exposure such as heat shock at 55°C–65°C for ~30 min (Silar 2020). Other notable stressors, including ultraviolet (UV) radiation, cold shock, and elevated concentrations of obnoxious materials, also affect ascospore germination. The germination of ascospores is triggered by several stressors mediated by cell membrane ligands, although the specific receptors are unknown. These stressors activate the ascospores through unknown mechanisms that involve the Mpk2 MAPK kinase pathway (Figure 3). Further, the Nox2/Pls1 NADPH oxidase is activated and the resultant effect of this complex activation is usually the production of reactive oxygen species (ROS), superoxide anions causing the breaching of the germinating pore by the germinating hyphae and thus germination of the ascospores. The accumulation of fungal peroxides often requires an NADPH oxidase and a MAP kinase cascade during fruiting body morphogenesis, including cell differentiation and cell degeneration/senescence, in most ascomycetes, such as *P. anserina* (Silar 2005). Worthy to note is that while the FPR1 gene, which is the only gene possessed by mat+ analogue, and the FMR1 gene from the mat‐ idiomorph of the sexual reproductive mating types (non‐homothallic) are both responsible for the recognition between sexual partners for fertilisation, the formation of ascospores is controlled by two genes notably SMR1 and SMR2 that are both found in the mat‐ strain in addition to FMR1. The mat+ only contains the FPR1 gene.

### 3.2 The Primordium Evolution

This follows immediately after the hyphae aggregations. The primordia, which are small, compact structures, quickly emerge from the aggregated hyphae, usually induced by hormonal and environmental stressors (Vonk and Ohm 2018). Most fruiting body formations are initiated when the mycelia (a complex network of interconnected hyphae) recognise specific nutrient depletion and a temperature downshift through specialised signalling molecules such as pheromones, causing a switch from vegetative growth to sexual reproductive development. Some transcription factors (TFs, Figure 3) and exposure to blue light trigger the formation of fruiting bodies. The expression of the *FMR1* gene is regulated by the network of HMG‐box transcription factors (TFs) and *FPR1* mating‐type genes, which in turn activate the PRE1/MFM and PRE2/MFP receptor pheromone genes, respectively. The homeodomain (HD‐TF) plays essential roles in mating and growth. Several HMG TFs have fruiting body functional peculiarities and phenotypic modifications. For instance, targeted knockouts of pah1, pah2, pah5, and pah7 in *P. anserina* altered perithecium morphology (Silar 2020); this is especially true for the pah2 and pah5 orthologs that cause the development of fruiting bodies without neck differentiation, facilitating faster spore dispersal (Vonk and Ohm 2018). The HMG‐box TFs regulate sexual reproduction during fruiting body development (Sun et al. 2023). The specific roles of Pah3, Pah4, and Pah7 analogues have not been reported, as the regulatory principles that mediate the spatiotemporal gene expression during fruiting body development remain largely unclear (Schumacher et al. 2018); however, they are suggested to offer complementary roles during perithecia maturity (Silar 2005). The formation of female sexual structures, the ascogonia coils, is the first stage of sexual development. This is enveloped by sterile hyphae forming a young fruiting body called protoperithecium that eventually evolves to perithecium (Schumacher et al. 2018), as shown in Figure 3. In addition to the HMG‐box TF regulations, there is the striatin‐interacting phosphates and kinases (STRIPAK) complex, which consists of six proteins that regulate signal transduction and fruiting body development. The STRIPAK interacts with the mitogen‐activated protein kinase (MAPK) Mgv1, a regulator of the cell wall integrity pathway that controls the phosphorylation and nuclear accumulation of MgV1, thereby directing fruiting body responses to extracellular stimuli. The STRIPAK orthologs, particularly PRO11, PRO22, PRO44, PRO45, and SmMOB3 components (Figure 3), regulate hyphal fusion (anastomosis), vegetative growth, and sexual development (Chen et al. 2023). Specifically, the histone chaperone gene anti‐silencing factor 1 (asf1) and transcription factor gene PRO44 play crucial roles in fruiting body formation during sexual development as both PRO44 and ASF1 assist in the regulation of gene expression in the nucleus; however, while the Pro44 proteins form homodimers and are actively expressed in the outer layers of the young and developing fruiting bodies at the transcriptional level, asf1 usually acts as a suppressor of genes that are weakly expressed during morphogenesis (Schumacher et al. 2018). Further, the homologue StrA, which acts as a scaffold protein, has also been linked to the endoplasmic reticulum (ER) and nuclear envelope in *Aspergillus nidulans* (Wang et al. 2010) during ascosporogenesis. The interaction of SCI1 and PRO22 through PRO11 is typically transient and closely regulated by unknown spatiotemporal signalling events (Reschka et al. 2018).

### 3.3 Mycelial Extension and Maturation

Upon establishing the initial stages (hyphae and primordia), the primordium develops and intensifies effective mycelial growth, leading to maturity. These phases are supported by potential changes in the magnitude of environmental requirements, e.g., nutrient uptake and metabolic processes. For instance, a change in carbon (IV) oxide (CO_2_) demand usually occurs as the CO_2_ affinity increases during the early stages of hyphae aggregation and primordia evolution, but decreases at this stage (Sakamoto 2018). A high carbon‐to‐nitrogen ratio (C: N) is crucial to initiate the fruiting body in the early stages of development, while later stages usually require low C: N. Specific functional TFs also encode the mycelial growth and fructification. Typical examples could be seen in the Ca^2+^‐calcineurin signalling pathway‐mediated C_2_H_2_ TF *PuCRZ1*, which regulates mannitol tolerance and mycelial growth in *P. umbellatus*, the significant expression of the zinc‐transcriptional regulator, Zn_2_Cys6, at the mycelial growth relative to other stages during fruiting body development in *G. lucidum*, and positive regulation of the active mycelial growth and cellulase gene *FfCEL6B* expression through the *FfCEL6B* promoter activation by the MYB and FfMYB15 in *F. fliformis* (Sun et al. 2023). Again, a previous study has also reported optimal colony growth and conidiation in *P. oxalicum* through an active regulation of the expression of the downstream TF, *BrlA*, by a homeodomain TF, PoHtf (Guo et al. 2021). These TFs regulate morphological differentiation, environmental stress sensitivity, and secondary metabolism (Calvo et al. 2024; Son et al. 2020). A previous study that evaluated stage‐specific biomarkers involved in mycelia (MY) transformation to the fruiting bodies (FB) in *F. velutipes* and *C. militaris* based on iTRAQ and integrative transcriptomic approaches, respectively, reported upregulation of several metabolites, including aldehyde dehydrogenase, FDS protein, cysteine peroxiredoxin, 2‐translation elongation factor 1‐alpha, pyruvate carboxylase, fatty acid synthase, aspartate aminotransferase, mitogen‐activated protein kinases (MAPKs), and heat‐shock protein, driving carbohydrate metabolism (Liu, Chang, et al. 2017; Liu, Chavez, et al. 2017; Thananusak et al. 2023). Similarly, in Smmob3 and pro11 in *S. macrospora*, the transcription of STRIPAK complex genes is usually upregulated during early and late sexual development (Bernhards and Pöggeler 2011).

The fruiting body morphogenesis encompasses the primordia development processes into a mature fruiting body (Perithecium). The hyphal knots gradually differentiate into distinct tissues, creating structures such as the cap, also known as the pileus; the stalk, also referred to as the stipe; and the gills, also called pores, which are spore‐producing structures. Furthermore, the developing fruiting body undergoes cellular specialisation, whereby cells in the cap create supportive tissues while cells in the gills gradually differentiate into the characteristic spore‐producing cells, such as asci in ascomycetes and basidia in basidiomycetes. The spore formation mechanisms include meiosis and mitosis, resulting in the formation of haploid spores. The complex processes, such as genetic, physiological, biochemical, cell differentiation, tissue formation, and structural development, are exacerbated. A proteomic assessment of the edible mushroom *L. edodes* revealed the upregulation of a few fruiting body‐specific proteins, including cyclohexanone monooxygenase, gamma‐glutamyl transpeptidase, and aspartic protease, produced via inducible pathways (Song et al. 2018). As for signal transduction pathways, complex signalling pathways (Lengeler et al. 2000; Martínez‐Soto and Ruiz‐Herrera 2017; Palmer and Horton 2006) potentially transmit environmental signals to initiate the activation of specific regulatory genes, including MAPK, cAMP‐PKA for cyclic AMP‐dependent protein kinase A, and the high‐osmolarity glycerol (HOG) pathways.

### 3.4 Spore Formation and Dissemination

The production and dispersal of spores are key goals of fruiting body development. An adaptive strategy against environmental stress involves physiological modifications, including characteristic pigmentation (i.e., melanin for protection against ultraviolet radiation) and the possession of enriched chitin and other polysaccharides. Again, fungal fruiting bodies can maximise sexual reproductive success by producing premature spores as an emergency adaptive response to abrupt shifts in environmental conditions. The spore dispersal mechanism is typically achieved through autolysis, resulting in senescence (Sakamoto 2018). The evolutionary progress of fruiting bodies is controlled by autophagy or auto‐degradation, allowing for substantial autolysis of complete tissues (Nagy et al. 2011). During fungal fruiting body senescence, the functional characteristics gradually deteriorate, resulting in either increased mortality rates or a decline in fecundity, as mostly observed in *P. anserina* , a model fungus for the molecular study of ageing (Silar 2020). It is influenced by chemical factors like nutrients (e.g., carbon and nitrogen) and physical stressors (e.g., light and temperature). In effect, fruiting bodies in high‐temperature biotopes are more liable to shorter lifespans. The dispersal mechanisms could be active, e.g., ballistospore discharge in basidiomycetes and forcible ejection (Sánchez‐García et al. 2020) based on Jack in the box theory/descriptive model following turgor pressure in ascomycetes, as shown in Figure 2; however, passive dispersal is induced by wind, water, and animals.

## 4 Fruiting Body Biotechnological Applications

Advances in fruiting body biotechnological applications have created new prospects, including agriculture and food production, medicine, bioactive compound production, and environmental sustainability. These fungal fruiting body biotechnological innovations drive innovation across various industries, providing sustainable and health‐promoting solutions to global challenges. The detailed descriptions of the principal areas where fruiting body biotechnology has tremendously revolutionised are as follows:

### 4.1 Agricultural and Food Biotechnology

Many compounds used as biocontrol agents against weeds, pests, and pathogens for commercial agriculture are produced by fruiting bodies, including *P. ostreatus* (Ocimati et al. 2021), *C. militaris* (Kryukov et al. 2014), *C. minitans* (Zeng et al. 2012), *B. bassiana*, (Islam et al. 2023), *Trichoderma* spp. (Poveda 2021; Yao et al. 2023), *M. anisopliae* (Ashraf et al. 2024; Zimmermann 1993), and *L. lecanii* (Zhou et al. 2020).

The fruiting bodies act as biocontrol agents, exhibiting characteristic modes of action against pests, which are generally described as mycoinsecticides or pesticides (Islam et al. 2023), as documented in Table 3. These compounds are suitable alternatives to chemically formulated pesticides owing to their target specificity and eco‐friendliness. The efficacy of the edible mushroom, *P. ostreatus*, in managing the *Fusarium* wilt of Bananas caused by the soil‐borne pathogen *F. oxysporum* has been reported (Ocimati et al. 2021). Kryukov et al. (2014) reported that *C. militaris* caused a dose‐dependent decrease in the survival of *L. decemlineata* with significantly delayed developmental time, moulting, and decreased enzyme activities. A study by Islam et al. (2023), which evaluated the effect of *B. bassiana* against cotton leafworm *S. litura*, reported the highest mortality rate (MR = 82%) after 7 days of treatment, confirming the biocontrol potential of the native isolate of *B. bassiana*. Besides its biocontrol potential, the fruiting body of *Trichoderma* spp. also promotes plant growth, improves the agrochemical ecosystem, enhances nutrient uptake efficiency, confers resistance, and achieves near 100% biocontrol effectiveness (Poveda 2021; Yao et al. 2023). Adequate soil health can be achieved through the fruiting bodies and their mycelial activities, mainly when the organic matter decomposes after fruiting body senescence, promoting nutrient cycling. Biotechnological innovations drive these associated processes involving soil health and crop sustainability. In the area of fruiting body biodiversity, biotechnological advances, such as in vitro culture techniques and cryopreservation, have significantly facilitated the conservation of fungal pure cultures, maintained biodiversity, and preserved fruiting bodies for sustainable bioproduction, thereby enabling a transition to a sustainable bioeconomy. In food biotechnology, the efficiency and scalability of edible mushroom cultivation have increased significantly due to advances in biotechnological approaches, leading to improved quality and yields. Fruiting bodies are excellent cell factories for the efficient production of recombinant animal proteins, considering their low feedstock demands, robust secretory pathways, and ability to undergo post‐translational protein modification in functional genomic studies (Jo et al. 2023). The tissue culture and liquid culture systems are notable methods that have helped to improve the propagation of mushroom strains. The development of functional foods via incorporating fruiting body‐produced bioactive compounds has increased globally. Nutraceuticals offer complementary health advantages to basic nutrition, including boosting immunity and conferring specific resistance against infections. Specifically, the extracts from *C. militaris* are used as suitable protein and carbohydrate supplements (Thananusak et al. 2023). Edible mushrooms are cultivated and consumed because they are naturally low in fat, cholesterol‐free, and rich in vital protein sources (Ghorai et al. 2009). Numerous hydrolytic enzymes and secondary metabolites of high industrial value secreted by fruiting bodies are utilised in food processing and refined fodder quality (Amara and El‐Baky 2023; Ghorai et al. 2009).

**TABLE 3:** Potential biocontrol agents produced by fungi that form fruiting bodies are promising and effective alternatives to chemical pesticides, considering their pest target specificity, sensitivity, and eco‐friendliness (Islam et al. 2023; Ocimati et al. 2021; Kryukov et al. 2014; Zeng et al. 2012; Poveda 2021; Yao et al. 2023; Ashraf et al. 2024; Zimmermann 1993; Zhou et al. 2020).

Biocontrol agent	Type of fungi	Fruiting body	Target pest	Mode of action
*Beauveria bassiana*	Entomopathogenic fungus	Develops conidiophores that produce conidia (asexual spores)	Active against a wide range of insect pests including aphids, whiteflies, and beetles	Spores’ attachment on the insect cuticle germinate, penetrate and consequently death then cycle is continued by sporulation
*Pleurotus ostreatus* ^a^	Saprophytic fungus	Produces characteristically large and edible fruiting bodies referred to as mushrooms	Shows vigorous activity against nematodes' degradation	Fungal mycelium produces toxins that potentially immobilise nematodes utilising them as reliable nutrient source following digested by the fungus
*Trichoderma* spp.	Mycoparasitic fungus	Produces conidia on conidiophores and chlamydospores in some species	Active against a wide range of soil‐borne plant pathogens including those causing root rot (e.g., Rhizoctonia, Pythium, Fusarium, etc.)	Suppresses plant pathogens through several mechanisms including mycoparasitism a direct attack on pathogens competition for nutrients and space, and the production of antimicrobial compounds
*Metarhizium anisopliae*	Entomopathogenic fungus	Produces conidia from conidiophores that emerge from the insect host fter death	Active against a variety of insects mosquitoes, termites, and locusts	Infects hosts through spores that penetrate the cuticle and proliferate within, eventually kills the host then, new spores emerge
*Cordyceps militaris*	Entomopathogenic fungus	Forms brightly‐coloured, club‐shaped fruiting bodies that produce ascospores	Infects a variety of insects, particularly caterpillars	Spores infect the insect hosts causing death and producing fruit bodies that emerge from the insects’ body releasing spores into the environment
*Lecanicillium lecanii*	Entomopathogenic fungus	Produces conidia from conidiophores that are used for infection	Controls soft‐bodied insects like aphids, whiteflies, and thrips	Infects the insect hosts by penetrating its cuticle, grows inside the body and eventually kills it, produces new conidia on the insect cadaver that spread to other insects
*Coniothyrium minitans* ^a^	Mycoparasitic fungus	Produces pycnidia, asexual fruiting bodies that contain conidia	Active against Sclerotinia sclerotiorum the causative agent of white moulds in crops like beans, canola, and lettuce	Parasitises the sclerotia, resting bodies of *S. sclerotiorum* deactivating the pathogens ability to infect crops

^a^ Represents the biocontrol agents that show a narrow spectrum of activity, i.e., they target only a specific pest, unlike others that exhibit a characteristic broad activity spectrum against a wide range of pests, offering natural and sustainable solutions for managing agricultural pests and diseases.

### 4.2 Production of Bioactive Compounds/Mushroom‐Based Materials

Many bioactive compounds, as presented in Table 4, possess significant medicinal qualities, including ergothioneine, polysaccharides, lovastatin, terpenoids, alkaloids, *β*‐glucan, γ‐aminobutyric acid (GABA), peptide, chitin, and chitosan (Hawar et al. 2023). Biotechnological innovations have revolutionised the extraction, purification, and scale‐up, significantly enhancing the production of these valuable compounds. Ergothioneine, a bioactive amino acid and potent antioxidant, helps control cardiovascular disease and neurological decline. Humans encode a specific ergothioneine transporter that enables cellular ergothioneine uptake from particular diets. It is naturally low in foods, but fruiting bodies of *N. crassa* and *A. oryzae* produce ergothioneine in enormous amounts. Specifically, a metabolic engineering strategy aimed at altering the molecular composition of mycoprotein to enhance its nutritional value and sensory qualities in *A. oryzae* has been reported to increase the production of ergothioneine (Maini Rekdal et al. 2024). The ergothioneine biosynthetic pathways in these fungi consist of two crucial enzymes, Egt1 and Egt2, that catalyse ergothioneine production from essential amino acids, including histidine, S‐adenosylmethionine, and cysteine. Bioengineering the haeme biosynthetic enzymes enhanced the engineered edible fungal mycelia biomass, thereby boosting the intracellular haeme level. This essential cofactor gives red meat its characteristic red colour and flavour during cooking, making it a reliable meat alternative (Maini Rekdal et al. 2024). The beta‐glucans from *Ganoderma* spp. are also renowned for their immune‐boosting potential, serving as both pharmaceuticals and nutraceuticals (Azi et al. 2024, 2023; Zhou et al. 2020). The formulations of bioengineered mycoprotein from enhanced haeme‐overproducing strains into their corresponding red meat substitutes often require minimal postharvest processing, suggesting significant prospects for industrial sustainability. Many sustainable mycelium‐based materials for commercial packaging, construction, and textiles are made from mycelia. The fruiting body biotechnology supports refining mycelium growth processes to produce cost‐effective, strong, sustainable, and biodegradable materials.

**TABLE 4:** Bioactive compounds produced by fungal fruiting bodies and their associated agricultural, medicinal, and food relevance.

Bioactive agent	Analogue	Fungal species	Benefit	References
Fatty acids	Unsaturated fatty acids (e.g., oleic, palmitoleic acids‐ cardiovascular and anti‐inflammatory effects oleic acid, linoleic acids, myristoleic acids)	*C. militaris*	Exhibit	Wu, Uchida, et al. (2023), Wu, Yang, et al. (2023)
Triterpenoids	Ganoderic acid	*G. lucidum*	Show anti‐inflammatory, antitumour, and hepato protective effects, contributing to the fungal medicinal properties	Azi et al. (2024)
Phenolics	Flavonoids and phenolic acids antioxidant properties, reduce oxidative stress and lower the risk of chronic diseases	*G. lucidum*	Exhibit	Azi et al. (2024)
Alkaloids	Psilocybin psychoactive effects and potential therapeutic applications in mental health treatment, particularly for depression and anxiety	*Psilocybe* spp	Trigger	Hawar et al. (2023)
Organic acids	Lactic and citric acids antimicrobial properties and act as preservatives in food products	Most fungi	Confer	Usman et al. (2024)
Antibiotics	Penicillin	*Penicillium* spp.	Possess significantly revolutionised medicine as it shows great potency in treating bacterial infections	Rousta et al. (2024)
Proteins and peptides	Antimicrobial peptides	Most fungi	Exhibit activity against bacteria, fungi and viruses, sustaining pharmaceuticals and food preservation	Hawar et al. (2023)
Sterols for vitamin D2 and potentially shows anti cancer properties	Ergosterol	*N. crassa* and *A. oryzae*	Precursor	Hawar et al. (2023)
Polysaccharides	Chitin and beta‐glucans and mannan	*N. crassa*	Exhibit antitumour effects and immune‐modulating properties while enhancing immune response	Hoeksma et al. (2019)
Essential oils	Terpenes and other VOCs	Most Asco‐/Basidiomycota	Confer antimicrobial, antifungal, and insecticidal properties, beneficial in food preservation and natural pest control	Liming et al. (2016)
STE	Mycotoxins	*A. flavus* and *A. parasiticus*	Acts as a biogenic precursor of aflatoxins B1 and G1, (i.e., AFB1and AFG1), respectively kills *Anopheles gambiae* mosquito larvae	Islam et al. (2023)

Abbreviations: *A. oryzae*, *Aspergillus oryzae*; *N. crassa*, *Neurospora crassa*; STE, sterigmatocystin; VOCs, volatile organic compounds.

Furthermore, three‐dimensional (3D) printing, involving fruiting body biomass and biofabrication, is utilised to create novel structures, including eco‐friendly building materials and clothing. Before their fruiting body development, most mushrooms secrete a liquid on the surface of their mycelia, which is harvested and used to produce bioactive compounds (Wu, Uchida, et al. 2023; Wu, Yang, et al. 2023). Fruiting bodies are a vibrant source of new antibiotics and antiviral agents. The initial screenings and mass production of antibiotics are enhanced, offering potential sustainable solutions to decipher the increasing antibiotic resistance and emerging viral infections globally (Hawar et al. 2023; Rousta et al. 2024). The structurally distinctive bioactive compounds of biological and pharmaceutical importance, including alkaloids, polyketides, peptides, lactones, and steroids, have also been isolated from fruiting bodies. These compounds are known to possess anti‐inflammatory, antioxidant, antibiotic, anticancer, antibacterial, antifungal, antiviral, and cytotoxic effects (Hawar et al. 2023; Hoeksma et al. 2019; Liming et al. 2016; Rousta et al. 2024; Usman et al. 2024). Similarly, Rousta et al. (2024) reported that Beta‐glucan and chitin are the most promising secondary metabolites from fruiting bodies used for cancer treatment. In addition, Usman et al. (2024) demonstrated that fruiting bodies increase biotic stress tolerance in medicinal plants by possessing genes that optimise secondary metabolite production.

### 4.3 Optimised Cultivation Strategies

*Biotechnological advances involving the **optimisation** of the fruiting bodies* cultivation strategies through controlled environment cultivations (CECs), including automated nutrient uptake monitoring, CO_2_ regulation, accurate humidity, and optimum temperature (Cohen et al. 2022), have shown tremendous improvements in the yield and quality (Madusanka et al. 2024), circumventing the barriers associated with seasonal species‐type scarcity and ensuring year‐round fruiting body cultivations through optimised indoor conditions. Fruiting bodies have also demonstrated a formidable force in substrate engineering, substantially reducing substrate limitations. Several modified and novel substrates, including agricultural and industrial waste products, have been developed to enhance the growth of fruiting bodies. Apart from substrate scale‐up for marginal yields, they also substantially contribute to the circular economy by recycling organic waste and aligning with global waste minimisation and utilisation campaigns, helping to address environmental pollution and climate change impacts (Attias et al. 2020; Soto‐Cruz et al. 1999). Following the increasing interest in this area, these innovations offer promising insights for various industries globally, thereby demystifying the unabridged potential of fruiting body biotechnology (Liming et al. 2016). However, substrate engineering, trace element impacts, and gene regulation challenges exist. Again, technological cost, particularly in developing and formulating improved substrates, is usually capital‐intensive. The process complexity also limits biotechnological adoptions, especially among the subsistent fruiting body producers, considering complex formulation procedures and the environmental uncertainties, most of which are usually difficult to control (Madusanka et al. 2024). Interestingly, potential actionable strategies for addressing these challenges, such as substrate engineering, trace element impacts, and gene regulation in the context of sustainable industrial bioproduction for fungal fruiting bodies in practical applications, are proposed as follows: (1) Bioreactor cultivation via solid‐state fermentation (SSF) or submerged culture with control systems to ensure consistent and scalable production. (2) Strain‐specific formulations through custom substrates and trace elements (e.g., Zn, Fe, Mn, Cu, Se, etc.) supplementations, tailored nutrient composition per species, maximising yield and bioactive compound output. (3) Sustainable substrate sourcing by prioritising the use of agricultural waste based on the circular economy models (e.g., straw, sawdust, bagasse etc.) after pretreatments, like physical (grinding), chemical (lime), and biological (enzymatic digestion), to improve digestibility, thereby simultaneously reducing production costs and mitigating environmental pollution due to agro‐waste accumulation. (4) Bioprocess automation, particularly involving sensors + artificial intelligence (AI) for real‐time regulation, thereby significantly minimising human error and improving sustainability. (5) Multi‐omics modelling, integrating data‐driven prediction of gene–environment interactions to enhance precision cultivation and strain design robustness.

### 4.4 Environmental and Health Biotechnology Applications

Environmental biotechnology, which involves the application of fruiting bodies and mycelia to depurate contaminated environments (e.g., soil, water, sludge), a process referred to as myco‐remediation, has gained considerable attention globally. The waste streams from food and beverage industries, as well as during crop harvests, including brewer's spent grains and corn residues, can be utilised by many fruiting bodies. Experimental screening or computational prediction of potential feedstock vastly improves the scalability of the fermentation process and substantially reduces waste minimisation costs (Jo et al. 2023). Several fungi, including *Aspergillus* spp., *Rhizopus* spp., *Fusarium* spp., *Acremonium* spp., *and Trichoderma* spp., have been utilised for the removal of a wide range of xenobiotics, such as pesticides, hydrocarbons, heavy metals, and microplastics. Specifically, *A. awamori* and *F. proliferatum* in a compost microbial consortium led to a total petroleum hydrocarbon removal efficiency of over 90% in a diesel hydrocarbon‐polluted soil within two months (Nwankwegu et al. 2016). Again, bioaugmentation studies involving a single strain of *R. arrhizus* in gasoline‐polluted soil resulted in 71.10% removal efficiency after 8 weeks of incubation, indicating effective bio‐restoration for subsequent cultivation (Nwankwegu and Onwosi 2017). Fruiting bodies possess robust enzyme cocktails and versatile metabolic machinery to utilise complex lignocellulosic biomass. The cytochrome P450 in fruiting bodies also increases their potential for bioremediation applications. Most catabolic enzymes have low substrate specificity, allowing the fruiting body to degrade structurally diverse chemicals, ranging from organic pollutants to plastics. Extracellular oxidoreductases, including laccases and peroxidases conserved within fruiting bodies, can efficiently degrade a wide range of contaminants such as organochlorines, explosives, EDCs (endocrine disrupting chemicals), BTEX (benzene, toluene, ethylbenzene, xylenes), PAHs (polycyclic aromatic hydrocarbons), MTBE (methyl‐tert‐butyl ether), phenols, and synthetic dyes (Jo et al. 2023). Most fruiting bodies also play crucial roles in carbon sequestration in soil and aquatic ecosystems, reducing greenhouse gas emissions and significantly complementing climate change mitigation efforts. They affect soil carbon pools and fluxes as they obtain carbon from their plant partners while transferring mineral nutrients in a symbiotic loop (Hawkins et al. 2023; Jeewani et al. 2021; Orsi et al. 2022). Moreover, fruiting bodies are also invaluable in health and medicine, particularly in immunomodulatory and anticancer biotechnology. Studies on the immunomodulatory and anticancer properties of bioactive compounds derived from fruiting bodies, especially those from *Ganoderma* sp. (Azi et al. 2024) and *Cordyceps* (Shweta et al. 2023), have been reported. For instance, *C. militaris*, an ascomycete, has proven to be highly relevant in traditional Chinese medicine due to the potential of its bioactive compounds, including polysaccharides, amino acids, mannitol, sterols, adenosine, and cordycepin. Essentially, cordycepin, a nucleoside derivative (3′‐deoxyadenosine), has garnered significant attention due to its therapeutic applications as an anti‐hyperlipidaemic, antiviral, immunomodulatory, anti‐inflammatory, anti‐diabetic, antitumour, anticancer, antimicrobial, and antifungal agent (Thananusak et al. 2023). Additionally, fruiting bodies are potential probiotics and prebiotics due to their richness in polysaccharides like hemicellulose, mannans, xylans, chitin, galactans, α‐ and *β*‐glucans (Singdevsachan et al. 2016). The probiotics and prebiotics derived from fruiting body products have demonstrated exceptional potency in the gut microbiome (Li et al. 2021; Ziemer and Gibson 1998), offering benefits to gut health. Although fruiting bodies are essential sources of probiotics and prebiotics, notable challenges have confronted their full‐scale adoption, including gut potency, overcoming biological constraints to survival, consumer acceptance, limitations associated with production scalability, and complex regulatory systems. Interestingly, advancements in fruiting body biotechnology have largely alleviated these limitations, driving the formulation and stability of the products.

### 4.5 Genetic Engineering and Synthetic Biology Applications

**FIGURE 4:**
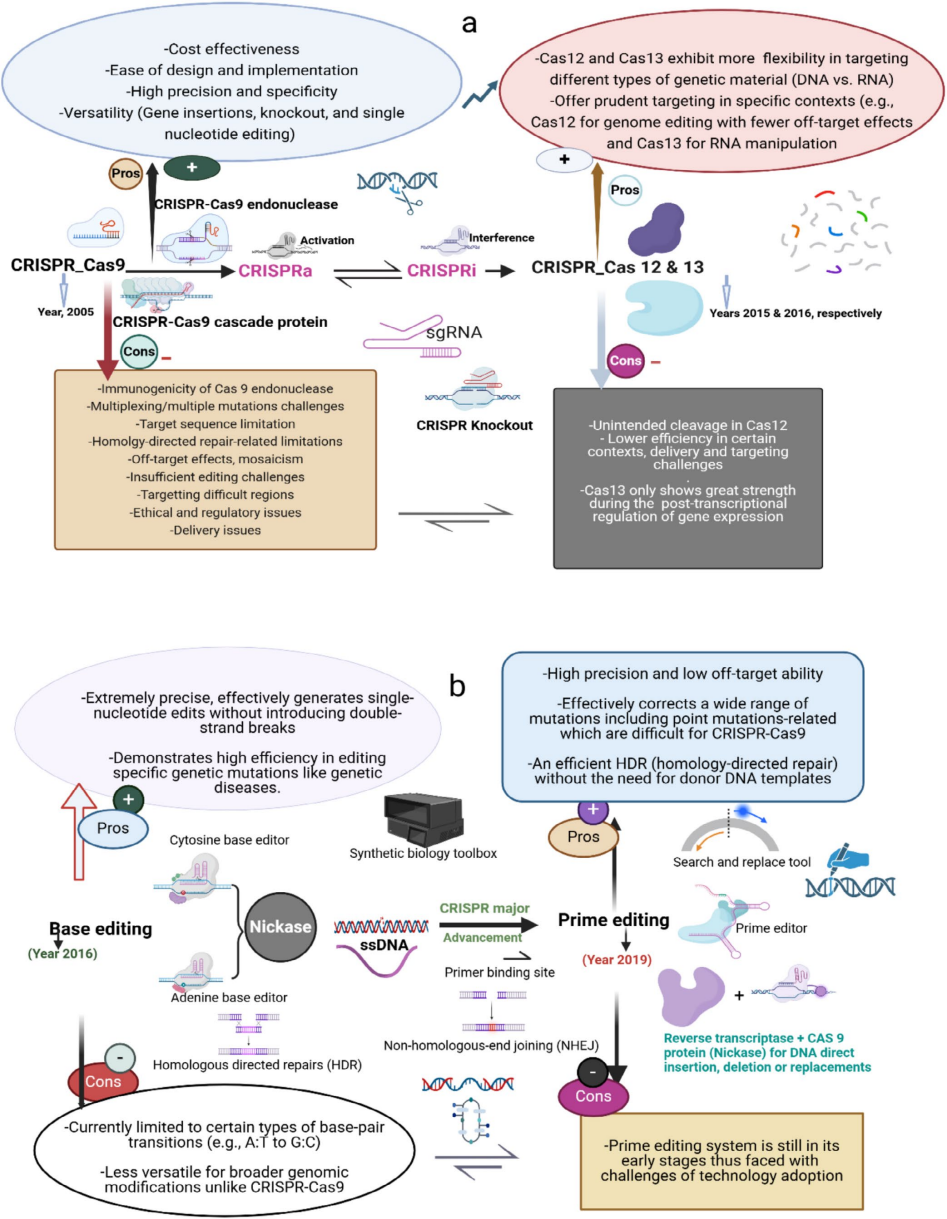
Synthetic biology toolbox for the genomic constructions, pathways, or organisms with custom‐designed properties describing CRISPR (a), CRISPR technological advancements (b), and their characteristic strengths (pros+) and inadequacies (cons‐). Directions of horizontal arrows represent technological advances, chronologically clarified by the corresponding years of discovery. Equal double half arrows interpret simultaneous CRISPR applications and relative technological equality; Unequal double arrows denote improved efficiency/decreased limitations following technological advancements.

Fungal evolution represents one of the Earth's most successful microbial entities, characterised by diverse morphologies and ecological functions (Merényi et al. 2023). In the industrial ecosystem, challenges related to slow mycelial growth, downstream purification, and low yields in strain development are addressed by a combination of biotechnological approaches, including computational modelling, experimental evolution, mutagenesis, and genetic engineering (Jo et al. 2023). The applications of fruiting bodies in genetic engineering, involving recombinant DNA technology (e.g., cloning and transgenic organisms) and synthetic biology (e.g., biological circuit designs and de novo DNA synthesis), have increased geometrically in recent years. While recombinant DNA (rDNA) technology has helped biologists to manipulate and combine DNA from different sources to create new DNA sequences that are not natural to the organisms' genome, synthetic biology, which consists of a broader and interdisciplinary area, has significantly assisted gene experts to effectively combine biology, computer science, and engineering to construct and design new biological systems, organisms, or components that are naturally not in existence. It transcends from modifying existing gene sequences to generating entirely new biological systems. The promise of synthetic biology in fungal‐derived food improvements as a useful genetic tool for robust applications in industrial food production has gained attention, helping to reduce planetary impacts due to resource‐intensive industrial animal agriculture (Maini Rekdal et al. 2024), promoting increased resource safety and efficiency, reduced environmental footprints, and guaranteeing a relatively precise control of production (Maini Rekdal et al. 2024). Synthetic biology provides novel genetic and computational tools, leveraging affordable genome sequencing, gene synthesis, genome editing (insertion, deletion, or substitution), and directed evolution technologies, to facilitate metabolic engineering and optimise the production of exogenous and native value‐added products, such as metabolites and proteins (Jo et al. 2023). The screening of the biochemical activities of fruiting bodies from natural products against drug targets is optimised by synthetic biology. Maini Rekdal et al. (2024) explored the possibilities of fungal synthetic biology and successfully engineered *A. oryzae* mycoprotein to overproduce the nutraceutical ergothioneine at levels significantly higher than seen in wild‐type mushrooms, demonstrating the first‐ever attempt to modify endogenous ergothioneine biosynthesis for mycoprotein production. Similarly, the application of synthetic biology in solid‐state fermentation (SSF) development involving *C. militaris* resulted in the overproduction of crucial metabolites (Thananusak et al. 2023). In recent years, fungal gene editing has optimised fruiting bodies through targeted mutations and genetic selection (Schuster and Kahmann 2019). The newer gene‐editing tool, such as the clustered regularly interspaced short palindromic repeats (CRISPR‐Cas9), has garnered tremendous attention in genome editing engineering, enabling selective DNA modifications tailored to human needs. The CRISPR‐Cas9 system (Figure 4) primarily involves the short synthetic single guide RNA (sgRNA) and the Cas9 endonucleases, known as molecular scissors, for DNA‐targeted cleavages. The Cas9 is from the aerotolerant and Gram‐positive bacterium *Streptococcus pyogenes*, commonly designated as SpCas‐9 (Schuster and Kahmann 2019). The CRISPR‐Cas9 system is promising as its editing permits multiplexing, i.e., multiple mutations (Foster et al. 2018; Schuster and Kahmann 2019) and potentially circumvents the inadequacies associated with the conventional DNA transfer/repair approaches, including homologous recombination usually characterised by double‐stranded breaks (DSBs), agrobacterium‐mediated integrations, and transposon tagging (Schuster and Kahmann 2019). The cloning technology, particularly modular Cloning (MoClo) based on Golden Gate assembly, provides the engineering chassis for constructing genetic libraries into multiple transcription units on a plasmid. Other DNA repair strategies in the context of synthetic biology cassette include nonhomologous end‐joining (NHEJ) for random DNA insertions, homologous directed repair (HDR) for editing target DNA from an exogenous template, accounting for precision, transposon sequence and mutagenesis (Tn‐Seq.) or transposon insertion sequence (TIS) for generating an extensive library. These can possess millions of mutants and have been proven faster than the traditional knockout approach (Jo et al. 2023). A high‐throughput gene knockout procedure involving the deletion of *mus‐51* or *mus‐52* genes that control nonhomologous end‐joining DNA repair, reduced ectopic integration and significantly (> 90%.) improved homologous recombination in *N. crassa* against its wild‐type with a low homologous recombination rate of < 10% (Park et al. 2013). Fruiting body genetic engineering and synthetic biology have profoundly enhanced the acquisition of desirable traits, such as increased disease resistance, accelerated growth rates, improved nutritional quality, critical environmental tolerance, and scalability of fungal‐derived food (Maini Rekdal et al. 2024). The CRISPR/Cas9‐mediated mutations are detected and analysed by Southern blotting, restriction enzyme analysis, and next‐generation sequencing (Cao et al. 2023; Saini et al. 2023). Azi et al. (2023) designed a synthetic consortium of *G. lucidum* and *L. plantarum* for enhanced natural product biosynthesis. They reported that small signalling molecules stimulated natural product biosynthesis by overexpressing target genes, which enhanced the secretion of ganoderic acids and exopolysaccharides. Again, Maini Rekdal et al. (2024) developed a modular synthetic biology toolkit based on the CRISPR‐Cas9 approach for *A. oryzae* and revealed elevated intracellular levels of the nutraceutical ergothioneine and the molecule haeme in the fruiting body biomass. However, recent advancements in CRISPR technology as shown in Figure 4, including optogenetics, prime and base editing, CRISPR‐Cas12 and CRISPR‐Cas13, CRISPRa, and CRISPRi (for activation and interference, respectively), have integrated newer genomic facilities with better delivery systems, more precise editing strategies, improved Cas9 variants, and wider public acceptance. They have largely addressed the CRISPR‐Cas9 shortcomings such as Cas9 immunogenicity, epigenetic modifications, limited target sequence, multiplexing challenges, targeting difficult regions, issues related to delivery, mosaicism, insufficient editing, challenges of homology‐directed repair, off‐target effects (OTEs), as well as ethical and regulatory concerns. The **f**ruiting body synthetic biology advances have also empowered metabolic pathway engineering for the synthesis of value‐added biomolecules. Specific fruiting body metabolic pathways are engineered to optimise the production of industrially relevant bioactive molecules, pigments, and enzymes (Afifa et al. 2022; Jo et al. 2023; Maini Rekdal et al. 2024; Peng et al. 2024). Further, mathematical and computational approaches involving constraint‐based and stoichiometric modelling are promising hotspots for metabolic engineering used for the fast translation of genomic information to predicted metabolic phenotypes so that genetic modifications are easily identified by silico biodiversity prospecting. Adoption of computational algorithms for metabolic engineering in fruiting bodies is a prudent approach for performance optimisation including improved native pathway synthesis, boosting end‐product yield, environmental control, and optimised media input (Jo et al. 2023).

## 5 Epigenetic Modifications Driving Fruiting Body Formation

**FIGURE 5:**
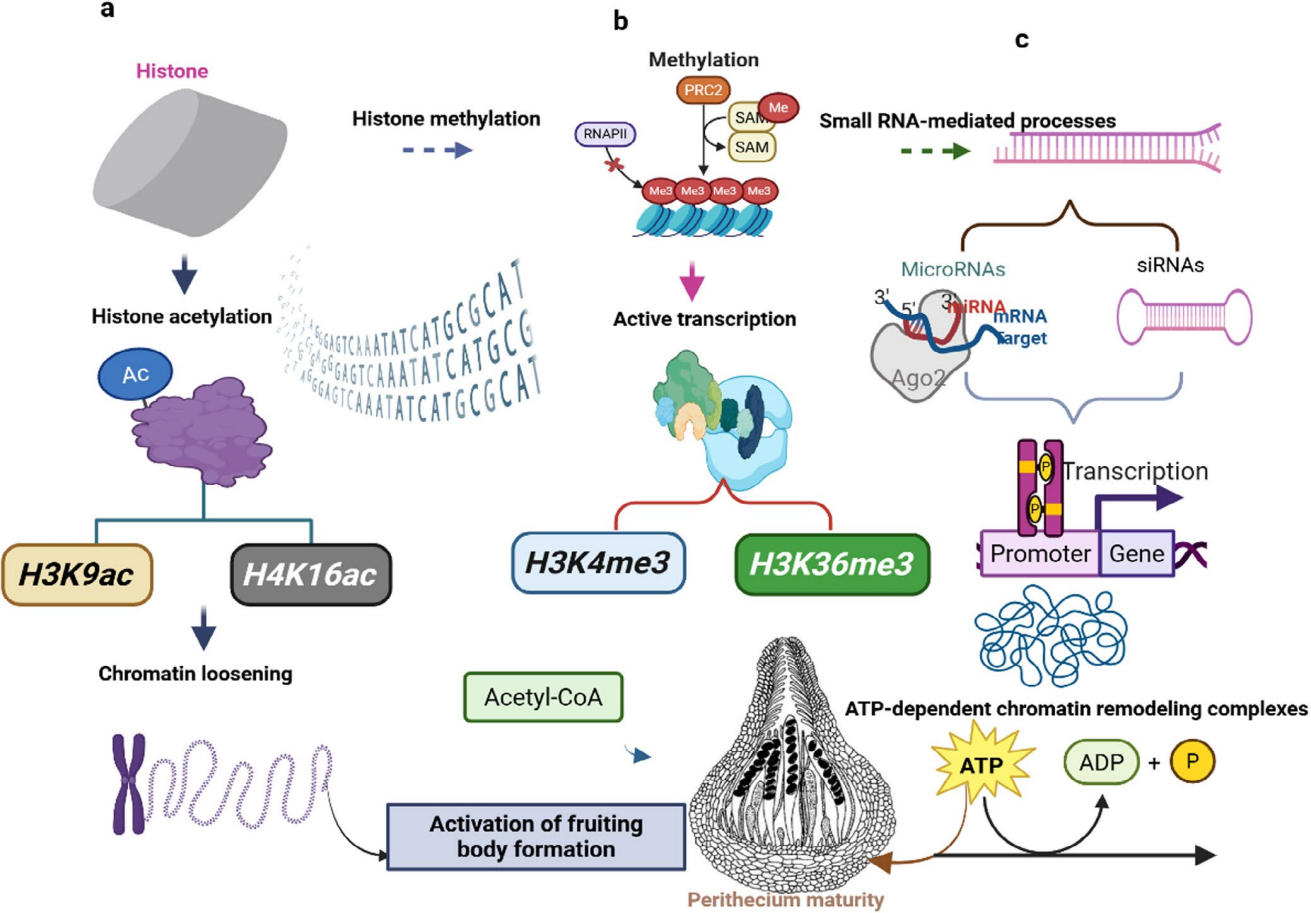
Potential epigenetic regulations driving fruiting body development and influencing gene expression patterns that regulate differentiation, morphogenesis, ascospore formation, and dispersal. (a) Histone modifications involving acylation (H3K9ac and H4K16ac). (b) Histone methylation (H3K4me3 and H3K36me3), and (c) small‐RNA‐mediated processes involving microRNAs and siRNAs.

Epigenetic regulation is crucial for fruiting body development, influencing gene expression patterns that regulate differentiation, morphogenesis, ascospore formation, and dispersal (Nowrousian 2022). Adequate knowledge of these mechanisms could potentially scale up fungal biotechnological applications, including optimising mushroom cultivation like *Ganoderma lucidum* for food and nutraceuticals (Azi et al. 2023) or controlling pathogenic fungi for the sustainable production of bioinsecticides, e.g., *Metarhizium anisopliae* (Zimmermann 1993). As presented in Figure 5, epigenetic modifications are essential for controlling fungal fruiting body development by regulating gene expression without altering the underlying DNA sequence. The potential epigenetic modifications include histone modifications, DNA methylation, and small‐RNA‐mediated processes (Etier et al. 2022). The histone modifications that involve histone acetylation (*H3K9ac* and *H4K16ac*) are often associated with transcriptional activation of genes required for fruiting body morphogenesis. Essentially, histone acetylation regulates gene expression by loosening chromatin structure, thereby facilitating transcription, and the histone acetylation marks, *H3K9ac* (histone H3 lysine 9 acetylation) and *H4K16ac* (histone H4 lysine 16 acetylation), perform critical roles in activating genes required for fungal fruiting body formation (Adhvaryu et al. 2005). Previous studies on *Schizophyllum commune* and *Neurospora crassa* suggest that dynamic histone modifications regulate genes involved in cell differentiation and sporulation (Aramayo and Selker 2013; Pelkmans et al. 2017). Histone methylation (*H3K4me3, H3K36me3*) is a marker of active chromatin regions, while repressive marks, such as H3K9me3 and H3K27me3, can silence developmental genes (Aramayo and Selker 2013). The histone H3 lysine 4 trimethylation (*H3K4me3*) and histone H3 lysine 36 trimethylation (*H3K36me3*) are two critical epigenetic markers associated with active transcription, influencing chromatin structure and gene activation to regulate precise developmental transitions during fruiting body formation. Small RNAs, including microRNAs and siRNAs, participate in post‐transcriptional regulation during fruiting body formation in RNA‐mediated epigenetic regulation. These RNA molecules can silence or activate genes involved in morphogenesis, stress response, and secondary metabolism. Moreover, ATP‐dependent chromatin remodelling complexes usually facilitate the transition between vegetative growth and fruiting body development (Nowrousian 2022; Vincent et al. 2009). Chromatin accessibility typically influences the transcription of key developmental regulators.

## 6 Concluding Remarks and Future Research Perspectives

This review extensively reports the critical environmental optima for fungal fruiting body development, particularly in regulating the multi‐omics footprint, including genomic, metabolomic, and proteomic perspectives, providing deep insights into the impacts of critical environmental optima on fruiting body molecular mechanisms. It revealed how transcription factors (TFs) regulate specific stages of fruiting body development; it critically examined CRISPR‐Cas9 as a powerful synthetic biology tool. It delineated the potential strengths and limitations of CRISPR‐Cas9, including the immunogenicity of the endonuclease (Cas9), ethical and regulatory issues, epigenetic modifications, limited target sequence, challenges associated with multiplexing, targeting difficult regions, insufficient editing, difficulties with homology‐directed repair, and off‐target effects. Furthermore, it chronologically synthesised the advancements in CRISPR technology, highlighting emerging technologies such as CRISPR‐Cas12 and CRISPR‐Cas13, CRISPRa and CRISPRi, optogenetics, prime editing, and base editing, which incorporate improved delivery systems, more precise editing strategies, and enhanced Cas9 variants. It also evaluated the fungal fruiting body epigenetic and developmental plasticity and explored how transcriptional and post‐translational modifications, including histone modification, DNA methylation, and small‐RNA‐mediated processes, influence the fruiting body in response to environmental heterogeneity. Finally, it recommends further studies on fruiting body biotechnology, especially those addressing the challenges associated with substrate engineering, including the cost and process complexity that tend to limit the commercial cultivation of fruiting bodies. It noted that addressing challenges such as substrate engineering, trace element impacts, and gene regulation is essential for achieving sustainable industrial bioproduction, stressing that multifaceted approaches, including system biology and bioprocess engineering, would be required to overcome them, specifically by combining biotechnology tools like CRISPR, omics, process optimisation, and cost‐effective/sustainable practices (e.g., AI‐driven control, waste reuse, use of agro‐industrial biomasses, like recyclable lignocellulosic waste to improve digestibility, **t**ailored nutrient composition, which addresses deficiency and toxicity of trace elements, supplementing substrates with carbon (e.g., molasses, starch), nitrogen (e.g., soybean meal, ammonium salts), and vitamins to suit species‐specific need, promoting industrial‐scale production of fungal fruiting bodies through more reliable, sustainable, and profitable strategies, thereby **s**upporting applications in food, pharmaceuticals, and green biotechnology. The study, therefore, offers promising insights for mycologists, researchers, and decision‐makers on enhancing fruiting body development for industrial bioproduction and transitioning to a sustainable bioeconomy.

## Author Contributions

**Amechi S. Nwankwegu:** conceptualization, investigation, writing – original draft, methodology, visualization, writing – review and editing, formal analysis, data curation. **Sinang Hongsanan:** visualization, funding acquisition. **Uzoma P. Nwankwegu:** formal analysis, visualization. **Ning Xie:** funding acquisition, supervision, writing – review and editing.

## Conflicts of Interest

The authors declare no conflicts of interest.

## Data Availability

The datasets generated during and/or analysed during the current study are available from the corresponding author on reasonable request.
